# Supplementary material for: Pyruvate Dehydrogenase Kinase 1 inhibition mediated oxidative phosphorylation enhancement in cartilage promotes osteoarthritis progression
Source: BMC Musculoskelet Disord. 2023 Jul 20;24:597. doi: 10.1186/s12891-023-06585-6 (PMC10357736; doi:10.1186/s12891-023-06585-6)
Supplement: Supplementary file 1 — Additional file 1. [file 12891_2023_6585_MOESM1_ESM.zip › KEGG.Gsea.1653622432464/KEGG_CYTOKINE_CYTOKINE_RECEPTOR_INTERACTION.html]

Details for gene set KEGG\_CYTOKINE\_CYTOKINE\_RECEPTOR\_INTERACTION[GSEA]

|  || Dataset | 55457\_collapsed\_to\_symbols.cls.cls #OA\_versus\_normal.cls.cls #OA\_versus\_normal\_repos |
| Phenotype | cls.cls#OA\_versus\_normal\_repos |
| Upregulated in class | normal |
| GeneSet | KEGG\_CYTOKINE\_CYTOKINE\_RECEPTOR\_INTERACTION |
| Enrichment Score (ES) | -0.21508698 |
| Normalized Enrichment Score (NES) | -1.0568979 |
| Nominal p-value | 0.32959327 |
| FDR q-value | 0.5881926 |
| FWER p-Value | 1.0 |
Table: GSEA Results Summary

  

Fig 1: Enrichment plot: KEGG\_CYTOKINE\_CYTOKINE\_RECEPTOR\_INTERACTION      
 Profile of the Running ES Score & Positions of GeneSet Members on the Rank Ordered List

  

| SYMBOL | TITLE | RANK IN GENE LIST | RANK METRIC SCORE | RUNNING ES | CORE ENRICHMENT || 1 | TNFRSF11B | TNF receptor superfamily member 11b [Source:HGNC Symbol;Acc:HGNC:11909] | 37 | 1.014 | 0.0122 | No |
| 2 | PDGFC | platelet derived growth factor C [Source:HGNC Symbol;Acc:HGNC:8801] | 103 | 0.911 | 0.0207 | No |
| 3 | CX3CR1 | C-X3-C motif chemokine receptor 1 [Source:HGNC Symbol;Acc:HGNC:2558] | 230 | 0.799 | 0.0227 | No |
| 4 | IL23A | interleukin 23 subunit alpha [Source:HGNC Symbol;Acc:HGNC:15488] | 312 | 0.740 | 0.0274 | No |
| 5 | IL17RB | interleukin 17 receptor B [Source:HGNC Symbol;Acc:HGNC:18015] | 399 | 0.707 | 0.0311 | No |
| 6 | IL11RA | interleukin 11 receptor subunit alpha [Source:HGNC Symbol;Acc:HGNC:5967] | 500 | 0.659 | 0.0331 | No |
| 7 | IL10RA | interleukin 10 receptor subunit alpha [Source:HGNC Symbol;Acc:HGNC:5964] | 588 | 0.628 | 0.0356 | No |
| 8 | IFNAR2 | interferon alpha and beta receptor subunit 2 [Source:HGNC Symbol;Acc:HGNC:5433] | 616 | 0.618 | 0.0427 | No |
| 9 | IL20RA | interleukin 20 receptor subunit alpha [Source:HGNC Symbol;Acc:HGNC:6003] | 668 | 0.601 | 0.0476 | No |
| 10 | CSF1R | colony stimulating factor 1 receptor [Source:HGNC Symbol;Acc:HGNC:2433] | 713 | 0.590 | 0.0530 | No |
| 11 | TNFSF13 | TNF superfamily member 13 [Source:HGNC Symbol;Acc:HGNC:11928] | 787 | 0.572 | 0.0557 | No |
| 12 | TNFRSF17 | TNF receptor superfamily member 17 [Source:HGNC Symbol;Acc:HGNC:11913] | 807 | 0.567 | 0.0627 | No |
| 13 | IFNGR2 | interferon gamma receptor 2 [Source:HGNC Symbol;Acc:HGNC:5440] | 861 | 0.552 | 0.0668 | No |
| 14 | FLT3LG | fms related receptor tyrosine kinase 3 ligand [Source:HGNC Symbol;Acc:HGNC:3766] | 894 | 0.544 | 0.0724 | No |
| 15 | IL21 | interleukin 21 [Source:HGNC Symbol;Acc:HGNC:6005] | 1199 | 0.477 | 0.0555 | No |
| 16 | IFNA8 | interferon alpha 8 [Source:HGNC Symbol;Acc:HGNC:5429] | 1206 | 0.475 | 0.0621 | No |
| 17 | TNFSF10 | TNF superfamily member 10 [Source:HGNC Symbol;Acc:HGNC:11925] | 1241 | 0.468 | 0.0664 | No |
| 18 | CXCL14 | C-X-C motif chemokine ligand 14 [Source:HGNC Symbol;Acc:HGNC:10640] | 1256 | 0.467 | 0.0723 | No |
| 19 | CD27 | CD27 molecule [Source:HGNC Symbol;Acc:HGNC:11922] | 1473 | 0.432 | 0.0617 | No |
| 20 | CXCL13 | C-X-C motif chemokine ligand 13 [Source:HGNC Symbol;Acc:HGNC:10639] | 1538 | 0.422 | 0.0630 | No |
| 21 | CXCL10 | C-X-C motif chemokine ligand 10 [Source:HGNC Symbol;Acc:HGNC:10637] | 1709 | 0.396 | 0.0555 | No |
| 22 | CCR2 | C-C motif chemokine receptor 2 [Source:HGNC Symbol;Acc:HGNC:1603] | 1815 | 0.382 | 0.0529 | No |
| 23 | IL12RB2 | interleukin 12 receptor subunit beta 2 [Source:HGNC Symbol;Acc:HGNC:5972] | 1857 | 0.379 | 0.0553 | No |
| 24 | CXCL6 | C-X-C motif chemokine ligand 6 [Source:HGNC Symbol;Acc:HGNC:10643] | 1916 | 0.370 | 0.0563 | No |
| 25 | TNFRSF11A | TNF receptor superfamily member 11a [Source:HGNC Symbol;Acc:HGNC:11908] | 1932 | 0.369 | 0.0606 | No |
| 26 | CCL19 | C-C motif chemokine ligand 19 [Source:HGNC Symbol;Acc:HGNC:10617] | 2060 | 0.355 | 0.0559 | No |
| 27 | CCR6 | C-C motif chemokine receptor 6 [Source:HGNC Symbol;Acc:HGNC:1607] | 2095 | 0.350 | 0.0584 | No |
| 28 | TNF | tumor necrosis factor [Source:HGNC Symbol;Acc:HGNC:11892] | 2098 | 0.350 | 0.0635 | No |
| 29 | KIT | "KIT proto-oncogene, receptor tyrosine kinase [Source:HGNC Symbol;Acc:HGNC:6342]" | 2117 | 0.348 | 0.0672 | No |
| 30 | TNFRSF21 | TNF receptor superfamily member 21 [Source:HGNC Symbol;Acc:HGNC:13469] | 2228 | 0.336 | 0.0636 | No |
| 31 | VEGFC | vascular endothelial growth factor C [Source:HGNC Symbol;Acc:HGNC:12682] | 2239 | 0.335 | 0.0678 | No |
| 32 | IL7 | interleukin 7 [Source:HGNC Symbol;Acc:HGNC:6023] | 2320 | 0.324 | 0.0663 | No |
| 33 | PLEKHO2 | pleckstrin homology domain containing O2 [Source:HGNC Symbol;Acc:HGNC:30026] | 2368 | 0.318 | 0.0674 | No |
| 34 | EPO | erythropoietin [Source:HGNC Symbol;Acc:HGNC:3415] | 2369 | 0.318 | 0.0721 | No |
| 35 | EGF | epidermal growth factor [Source:HGNC Symbol;Acc:HGNC:3229] | 2399 | 0.315 | 0.0745 | No |
| 36 | IL10RB | interleukin 10 receptor subunit beta [Source:HGNC Symbol;Acc:HGNC:5965] | 2438 | 0.309 | 0.0761 | No |
| 37 | GH1 | growth hormone 1 [Source:HGNC Symbol;Acc:HGNC:4261] | 2456 | 0.308 | 0.0794 | No |
| 38 | IL18 | interleukin 18 [Source:HGNC Symbol;Acc:HGNC:5986] | 2472 | 0.306 | 0.0828 | No |
| 39 | TGFB1 | transforming growth factor beta 1 [Source:HGNC Symbol;Acc:HGNC:11766] | 2490 | 0.303 | 0.0860 | No |
| 40 | HGF | hepatocyte growth factor [Source:HGNC Symbol;Acc:HGNC:4893] | 2547 | 0.297 | 0.0860 | No |
| 41 | TNFSF11 | TNF superfamily member 11 [Source:HGNC Symbol;Acc:HGNC:11926] | 2557 | 0.297 | 0.0897 | No |
| 42 | IL15 | interleukin 15 [Source:HGNC Symbol;Acc:HGNC:5977] | 2599 | 0.291 | 0.0908 | No |
| 43 | CCR5 | C-C motif chemokine receptor 5 [Source:HGNC Symbol;Acc:HGNC:1606] | 2617 | 0.289 | 0.0938 | No |
| 44 | BMPR1A | bone morphogenetic protein receptor type 1A [Source:HGNC Symbol;Acc:HGNC:1076] | 2745 | 0.275 | 0.0879 | No |
| 45 | CCR3 | C-C motif chemokine receptor 3 [Source:HGNC Symbol;Acc:HGNC:1604] | 2773 | 0.273 | 0.0898 | No |
| 46 | TNFRSF14 | TNF receptor superfamily member 14 [Source:HGNC Symbol;Acc:HGNC:11912] | 2940 | 0.257 | 0.0805 | No |
| 47 | PDGFRA | platelet derived growth factor receptor alpha [Source:HGNC Symbol;Acc:HGNC:8803] | 2943 | 0.256 | 0.0842 | No |
| 48 | CXCR6 | C-X-C motif chemokine receptor 6 [Source:HGNC Symbol;Acc:HGNC:16647] | 3045 | 0.245 | 0.0799 | No |
| 49 | TNFSF8 | TNF superfamily member 8 [Source:HGNC Symbol;Acc:HGNC:11938] | 3107 | 0.239 | 0.0787 | No |
| 50 | CCL13 | C-C motif chemokine ligand 13 [Source:HGNC Symbol;Acc:HGNC:10611] | 3379 | 0.214 | 0.0605 | No |
| 51 | CXCR5 | C-X-C motif chemokine receptor 5 [Source:HGNC Symbol;Acc:HGNC:1060] | 3397 | 0.212 | 0.0623 | No |
| 52 | CCR9 | C-C motif chemokine receptor 9 [Source:HGNC Symbol;Acc:HGNC:1610] | 3421 | 0.209 | 0.0636 | No |
| 53 | CCL27 | C-C motif chemokine ligand 27 [Source:HGNC Symbol;Acc:HGNC:10626] | 3495 | 0.203 | 0.0609 | No |
| 54 | CXCL11 | C-X-C motif chemokine ligand 11 [Source:HGNC Symbol;Acc:HGNC:10638] | 3575 | 0.196 | 0.0576 | No |
| 55 | IFNAR1 | interferon alpha and beta receptor subunit 1 [Source:HGNC Symbol;Acc:HGNC:5432] | 3587 | 0.195 | 0.0596 | No |
| 56 | CSF2 | colony stimulating factor 2 [Source:HGNC Symbol;Acc:HGNC:2434] | 3699 | 0.185 | 0.0536 | No |
| 57 | XCL1 | X-C motif chemokine ligand 1 [Source:HGNC Symbol;Acc:HGNC:10645] | 3789 | 0.178 | 0.0492 | No |
| 58 | CSF2RA | colony stimulating factor 2 receptor subunit alpha [Source:HGNC Symbol;Acc:HGNC:2435] | 3797 | 0.178 | 0.0513 | No |
| 59 | FAS | Fas cell surface death receptor [Source:HGNC Symbol;Acc:HGNC:11920] | 3828 | 0.173 | 0.0516 | No |
| 60 | ACVR1 | activin A receptor type 1 [Source:HGNC Symbol;Acc:HGNC:171] | 3898 | 0.165 | 0.0486 | No |
| 61 | FASLG | Fas ligand [Source:HGNC Symbol;Acc:HGNC:11936] | 3931 | 0.162 | 0.0485 | No |
| 62 | CTF1 | cardiotrophin 1 [Source:HGNC Symbol;Acc:HGNC:2499] | 4064 | 0.150 | 0.0403 | No |
| 63 | IL9R | interleukin 9 receptor [Source:HGNC Symbol;Acc:HGNC:6030] | 4118 | 0.144 | 0.0383 | No |
| 64 | NGFR | nerve growth factor receptor [Source:HGNC Symbol;Acc:HGNC:7809] | 4156 | 0.142 | 0.0375 | No |
| 65 | LTB | lymphotoxin beta [Source:HGNC Symbol;Acc:HGNC:6711] | 4209 | 0.137 | 0.0354 | No |
| 66 | IFNA4 | interferon alpha 4 [Source:HGNC Symbol;Acc:HGNC:5425] | 4282 | 0.129 | 0.0316 | No |
| 67 | IL2RA | interleukin 2 receptor subunit alpha [Source:HGNC Symbol;Acc:HGNC:6008] | 4292 | 0.129 | 0.0328 | No |
| 68 | PRLR | prolactin receptor [Source:HGNC Symbol;Acc:HGNC:9446] | 4323 | 0.126 | 0.0324 | No |
| 69 | IL11 | interleukin 11 [Source:HGNC Symbol;Acc:HGNC:5966] | 4327 | 0.126 | 0.0340 | No |
| 70 | TNFRSF4 | TNF receptor superfamily member 4 [Source:HGNC Symbol;Acc:HGNC:11918] | 4383 | 0.122 | 0.0315 | No |
| 71 | TNFSF9 | TNF superfamily member 9 [Source:HGNC Symbol;Acc:HGNC:11939] | 4391 | 0.122 | 0.0328 | No |
| 72 | CCR10 | C-C motif chemokine receptor 10 [Source:HGNC Symbol;Acc:HGNC:4474] | 4458 | 0.117 | 0.0293 | No |
| 73 | PF4V1 | platelet factor 4 variant 1 [Source:HGNC Symbol;Acc:HGNC:8862] | 4522 | 0.112 | 0.0260 | No |
| 74 | EPOR | erythropoietin receptor [Source:HGNC Symbol;Acc:HGNC:3416] | 4596 | 0.105 | 0.0218 | No |
| 75 | FLT3 | fms related receptor tyrosine kinase 3 [Source:HGNC Symbol;Acc:HGNC:3765] | 4657 | 0.099 | 0.0185 | No |
| 76 | CCL18 | C-C motif chemokine ligand 18 [Source:HGNC Symbol;Acc:HGNC:10616] | 4659 | 0.099 | 0.0199 | No |
| 77 | INHBC | inhibin subunit beta C [Source:HGNC Symbol;Acc:HGNC:6068] | 4788 | 0.088 | 0.0112 | No |
| 78 | IL21R | interleukin 21 receptor [Source:HGNC Symbol;Acc:HGNC:6006] | 4810 | 0.087 | 0.0108 | No |
| 79 | IL2RG | interleukin 2 receptor subunit gamma [Source:HGNC Symbol;Acc:HGNC:6010] | 4887 | 0.081 | 0.0060 | No |
| 80 | CSF3 | colony stimulating factor 3 [Source:HGNC Symbol;Acc:HGNC:2438] | 4995 | 0.073 | -0.0013 | No |
| 81 | AMH | anti-Mullerian hormone [Source:HGNC Symbol;Acc:HGNC:464] | 5042 | 0.070 | -0.0039 | No |
| 82 | IL2RB | interleukin 2 receptor subunit beta [Source:HGNC Symbol;Acc:HGNC:6009] | 5045 | 0.069 | -0.0031 | No |
| 83 | CCR4 | C-C motif chemokine receptor 4 [Source:HGNC Symbol;Acc:HGNC:1605] | 5108 | 0.063 | -0.0070 | No |
| 84 | CCR1 | C-C motif chemokine receptor 1 [Source:HGNC Symbol;Acc:HGNC:1602] | 5110 | 0.063 | -0.0061 | No |
| 85 | CNTF | ciliary neurotrophic factor [Source:HGNC Symbol;Acc:HGNC:2169] | 5165 | 0.059 | -0.0095 | No |
| 86 | TNFRSF6B | TNF receptor superfamily member 6b [Source:HGNC Symbol;Acc:HGNC:11921] | 5168 | 0.058 | -0.0088 | No |
| 87 | IL5 | interleukin 5 [Source:HGNC Symbol;Acc:HGNC:6016] | 5277 | 0.050 | -0.0166 | No |
| 88 | TGFBR1 | transforming growth factor beta receptor 1 [Source:HGNC Symbol;Acc:HGNC:11772] | 5280 | 0.050 | -0.0160 | No |
| 89 | PRL | prolactin [Source:HGNC Symbol;Acc:HGNC:9445] | 5490 | 0.033 | -0.0320 | No |
| 90 | MPL | "MPL proto-oncogene, thrombopoietin receptor [Source:HGNC Symbol;Acc:HGNC:7217]" | 5508 | 0.032 | -0.0329 | No |
| 91 | IL24 | interleukin 24 [Source:HGNC Symbol;Acc:HGNC:11346] | 5517 | 0.031 | -0.0331 | No |
| 92 | CD40LG | CD40 ligand [Source:HGNC Symbol;Acc:HGNC:11935] | 5529 | 0.030 | -0.0335 | No |
| 93 | CCR7 | C-C motif chemokine receptor 7 [Source:HGNC Symbol;Acc:HGNC:1608] | 5678 | 0.017 | -0.0449 | No |
| 94 | IL17A | interleukin 17A [Source:HGNC Symbol;Acc:HGNC:5981] | 5758 | 0.011 | -0.0510 | No |
| 95 | CX3CL1 | C-X3-C motif chemokine ligand 1 [Source:HGNC Symbol;Acc:HGNC:10647] | 5764 | 0.011 | -0.0512 | No |
| 96 | IL2 | interleukin 2 [Source:HGNC Symbol;Acc:HGNC:6001] | 5819 | 0.007 | -0.0553 | No |
| 97 | IFNA2 | interferon alpha 2 [Source:HGNC Symbol;Acc:HGNC:5423] | 5869 | 0.004 | -0.0592 | No |
| 98 | TNFSF4 | TNF superfamily member 4 [Source:HGNC Symbol;Acc:HGNC:11934] | 5930 | 0.000 | -0.0639 | No |
| 99 | PF4 | platelet factor 4 [Source:HGNC Symbol;Acc:HGNC:8861] | 5937 | -0.000 | -0.0644 | No |
| 100 | BMPR1B | bone morphogenetic protein receptor type 1B [Source:HGNC Symbol;Acc:HGNC:1077] | 5959 | -0.002 | -0.0660 | No |
| 101 | ACVR2A | activin A receptor type 2A [Source:HGNC Symbol;Acc:HGNC:173] | 5960 | -0.002 | -0.0660 | No |
| 102 | IL12B | interleukin 12B [Source:HGNC Symbol;Acc:HGNC:5970] | 5991 | -0.004 | -0.0683 | No |
| 103 | IFNA21 | interferon alpha 21 [Source:HGNC Symbol;Acc:HGNC:5424] | 6127 | -0.015 | -0.0787 | No |
| 104 | GDF5 | growth differentiation factor 5 [Source:HGNC Symbol;Acc:HGNC:4220] | 6205 | -0.021 | -0.0845 | No |
| 105 | TGFB3 | transforming growth factor beta 3 [Source:HGNC Symbol;Acc:HGNC:11769] | 6293 | -0.027 | -0.0909 | No |
| 106 | TNFSF18 | TNF superfamily member 18 [Source:HGNC Symbol;Acc:HGNC:11932] | 6347 | -0.031 | -0.0947 | No |
| 107 | ACVR1B | activin A receptor type 1B [Source:HGNC Symbol;Acc:HGNC:172] | 6368 | -0.033 | -0.0957 | No |
| 108 | CXCR4 | C-X-C motif chemokine receptor 4 [Source:HGNC Symbol;Acc:HGNC:2561] | 6382 | -0.034 | -0.0963 | No |
| 109 | IFNG | interferon gamma [Source:HGNC Symbol;Acc:HGNC:5438] | 6410 | -0.036 | -0.0979 | No |
| 110 | IL26 | interleukin 26 [Source:HGNC Symbol;Acc:HGNC:17119] | 6419 | -0.037 | -0.0979 | No |
| 111 | CCL1 | C-C motif chemokine ligand 1 [Source:HGNC Symbol;Acc:HGNC:10609] | 6462 | -0.039 | -0.1007 | No |
| 112 | TPO | thyroid peroxidase [Source:HGNC Symbol;Acc:HGNC:12015] | 6467 | -0.040 | -0.1004 | No |
| 113 | CCL5 | C-C motif chemokine ligand 5 [Source:HGNC Symbol;Acc:HGNC:10632] | 6584 | -0.048 | -0.1088 | No |
| 114 | IL4 | interleukin 4 [Source:HGNC Symbol;Acc:HGNC:6014] | 6673 | -0.054 | -0.1150 | No |
| 115 | IFNA10 | interferon alpha 10 [Source:HGNC Symbol;Acc:HGNC:5418] | 6682 | -0.054 | -0.1148 | No |
| 116 | GHR | growth hormone receptor [Source:HGNC Symbol;Acc:HGNC:4263] | 6740 | -0.058 | -0.1184 | No |
| 117 | IFNA5 | interferon alpha 5 [Source:HGNC Symbol;Acc:HGNC:5426] | 6755 | -0.059 | -0.1186 | No |
| 118 | CD40 | CD40 molecule [Source:HGNC Symbol;Acc:HGNC:11919] | 6825 | -0.065 | -0.1231 | No |
| 119 | CCL21 | C-C motif chemokine ligand 21 [Source:HGNC Symbol;Acc:HGNC:10620] | 6856 | -0.068 | -0.1245 | No |
| 120 | CCL11 | C-C motif chemokine ligand 11 [Source:HGNC Symbol;Acc:HGNC:10610] | 6907 | -0.073 | -0.1273 | No |
| 121 | PPBP | pro-platelet basic protein [Source:HGNC Symbol;Acc:HGNC:9240] | 6910 | -0.073 | -0.1264 | No |
| 122 | PDGFRB | platelet derived growth factor receptor beta [Source:HGNC Symbol;Acc:HGNC:8804] | 6914 | -0.073 | -0.1255 | No |
| 123 | IL12A | interleukin 12A [Source:HGNC Symbol;Acc:HGNC:5969] | 6930 | -0.074 | -0.1256 | No |
| 124 | TGFB2 | transforming growth factor beta 2 [Source:HGNC Symbol;Acc:HGNC:11768] | 6985 | -0.079 | -0.1287 | No |
| 125 | GH2 | growth hormone 2 [Source:HGNC Symbol;Acc:HGNC:4262] | 6993 | -0.079 | -0.1281 | No |
| 126 | CXCL9 | C-X-C motif chemokine ligand 9 [Source:HGNC Symbol;Acc:HGNC:7098] | 7024 | -0.082 | -0.1292 | No |
| 127 | IFNGR1 | interferon gamma receptor 1 [Source:HGNC Symbol;Acc:HGNC:5439] | 7176 | -0.094 | -0.1397 | No |
| 128 | CRLF2 | cytokine receptor like factor 2 [Source:HGNC Symbol;Acc:HGNC:14281] | 7254 | -0.100 | -0.1443 | No |
| 129 | IFNA6 | interferon alpha 6 [Source:HGNC Symbol;Acc:HGNC:5427] | 7272 | -0.101 | -0.1442 | No |
| 130 | EDAR | ectodysplasin A receptor [Source:HGNC Symbol;Acc:HGNC:2895] | 7290 | -0.102 | -0.1440 | No |
| 131 | MET | "MET proto-oncogene, receptor tyrosine kinase [Source:HGNC Symbol;Acc:HGNC:7029]" | 7322 | -0.104 | -0.1449 | No |
| 132 | CD70 | CD70 molecule [Source:HGNC Symbol;Acc:HGNC:11937] | 7491 | -0.118 | -0.1564 | No |
| 133 | LIFR | LIF receptor subunit alpha [Source:HGNC Symbol;Acc:HGNC:6597] | 7625 | -0.129 | -0.1650 | No |
| 134 | INHBE | inhibin subunit beta E [Source:HGNC Symbol;Acc:HGNC:24029] | 7693 | -0.134 | -0.1683 | No |
| 135 | CSF2RB | colony stimulating factor 2 receptor subunit beta [Source:HGNC Symbol;Acc:HGNC:2436] | 7826 | -0.143 | -0.1765 | No |
| 136 | IL1A | interleukin 1 alpha [Source:HGNC Symbol;Acc:HGNC:5991] | 7842 | -0.144 | -0.1756 | No |
| 137 | IL22 | interleukin 22 [Source:HGNC Symbol;Acc:HGNC:14900] | 7997 | -0.154 | -0.1854 | No |
| 138 | IL13RA1 | interleukin 13 receptor subunit alpha 1 [Source:HGNC Symbol;Acc:HGNC:5974] | 8085 | -0.162 | -0.1899 | No |
| 139 | TNFSF15 | TNF superfamily member 15 [Source:HGNC Symbol;Acc:HGNC:11931] | 8093 | -0.163 | -0.1880 | No |
| 140 | IL22RA1 | interleukin 22 receptor subunit alpha 1 [Source:HGNC Symbol;Acc:HGNC:13700] | 8101 | -0.163 | -0.1861 | No |
| 141 | IL25 | interleukin 25 [Source:HGNC Symbol;Acc:HGNC:13765] | 8121 | -0.164 | -0.1852 | No |
| 142 | TNFRSF1A | TNF receptor superfamily member 1A [Source:HGNC Symbol;Acc:HGNC:11916] | 8196 | -0.171 | -0.1885 | No |
| 143 | CCL14 | C-C motif chemokine ligand 14 [Source:HGNC Symbol;Acc:HGNC:10612] | 8239 | -0.174 | -0.1892 | No |
| 144 | TGFBR2 | transforming growth factor beta receptor 2 [Source:HGNC Symbol;Acc:HGNC:11773] | 8241 | -0.174 | -0.1867 | No |
| 145 | CCL7 | C-C motif chemokine ligand 7 [Source:HGNC Symbol;Acc:HGNC:10634] | 8351 | -0.182 | -0.1925 | No |
| 146 | EDA2R | ectodysplasin A2 receptor [Source:HGNC Symbol;Acc:HGNC:17756] | 8364 | -0.183 | -0.1908 | No |
| 147 | LEPR | leptin receptor [Source:HGNC Symbol;Acc:HGNC:6554] | 8479 | -0.192 | -0.1969 | No |
| 148 | CCR8 | C-C motif chemokine receptor 8 [Source:HGNC Symbol;Acc:HGNC:1609] | 8497 | -0.194 | -0.1953 | No |
| 149 | IFNA7 | interferon alpha 7 [Source:HGNC Symbol;Acc:HGNC:5428] | 8533 | -0.196 | -0.1952 | No |
| 150 | IFNA1 | interferon alpha 1 [Source:HGNC Symbol;Acc:HGNC:5417] | 8640 | -0.204 | -0.2005 | No |
| 151 | TNFRSF13B | TNF receptor superfamily member 13B [Source:HGNC Symbol;Acc:HGNC:18153] | 8664 | -0.206 | -0.1992 | No |
| 152 | VEGFB | vascular endothelial growth factor B [Source:HGNC Symbol;Acc:HGNC:12681] | 8673 | -0.206 | -0.1968 | No |
| 153 | LEP | leptin [Source:HGNC Symbol;Acc:HGNC:6553] | 8740 | -0.212 | -0.1988 | No |
| 154 | TNFRSF8 | TNF receptor superfamily member 8 [Source:HGNC Symbol;Acc:HGNC:11923] | 8742 | -0.212 | -0.1957 | No |
| 155 | LTBR | lymphotoxin beta receptor [Source:HGNC Symbol;Acc:HGNC:6718] | 8743 | -0.212 | -0.1926 | No |
| 156 | IL12RB1 | interleukin 12 receptor subunit beta 1 [Source:HGNC Symbol;Acc:HGNC:5971] | 8768 | -0.213 | -0.1913 | No |
| 157 | CCL24 | C-C motif chemokine ligand 24 [Source:HGNC Symbol;Acc:HGNC:10623] | 8833 | -0.220 | -0.1930 | No |
| 158 | IL10 | interleukin 10 [Source:HGNC Symbol;Acc:HGNC:5962] | 8855 | -0.221 | -0.1914 | No |
| 159 | CXCR3 | C-X-C motif chemokine receptor 3 [Source:HGNC Symbol;Acc:HGNC:4540] | 9071 | -0.237 | -0.2048 | No |
| 160 | IL17RA | interleukin 17 receptor A [Source:HGNC Symbol;Acc:HGNC:5985] | 9202 | -0.248 | -0.2114 | Yes |
| 161 | IL5RA | interleukin 5 receptor subunit alpha [Source:HGNC Symbol;Acc:HGNC:6017] | 9204 | -0.248 | -0.2078 | Yes |
| 162 | IL7R | interleukin 7 receptor [Source:HGNC Symbol;Acc:HGNC:6024] | 9233 | -0.251 | -0.2062 | Yes |
| 163 | CSF3R | colony stimulating factor 3 receptor [Source:HGNC Symbol;Acc:HGNC:2439] | 9247 | -0.252 | -0.2035 | Yes |
| 164 | CCL15 | C-C motif chemokine ligand 15 [Source:HGNC Symbol;Acc:HGNC:10613] | 9354 | -0.260 | -0.2080 | Yes |
| 165 | XCR1 | X-C motif chemokine receptor 1 [Source:HGNC Symbol;Acc:HGNC:1625] | 9373 | -0.262 | -0.2055 | Yes |
| 166 | IL1RAP | interleukin 1 receptor accessory protein [Source:HGNC Symbol;Acc:HGNC:5995] | 9395 | -0.263 | -0.2032 | Yes |
| 167 | CXCL5 | C-X-C motif chemokine ligand 5 [Source:HGNC Symbol;Acc:HGNC:10642] | 9489 | -0.273 | -0.2065 | Yes |
| 168 | CCL22 | C-C motif chemokine ligand 22 [Source:HGNC Symbol;Acc:HGNC:10621] | 9496 | -0.274 | -0.2029 | Yes |
| 169 | CXCL1 | C-X-C motif chemokine ligand 1 [Source:HGNC Symbol;Acc:HGNC:4602] | 9528 | -0.276 | -0.2012 | Yes |
| 170 | BMP7 | bone morphogenetic protein 7 [Source:HGNC Symbol;Acc:HGNC:1074] | 9544 | -0.278 | -0.1982 | Yes |
| 171 | CCL23 | C-C motif chemokine ligand 23 [Source:HGNC Symbol;Acc:HGNC:10622] | 9610 | -0.284 | -0.1991 | Yes |
| 172 | CSF1 | colony stimulating factor 1 [Source:HGNC Symbol;Acc:HGNC:2432] | 9613 | -0.284 | -0.1950 | Yes |
| 173 | CXCR2 | C-X-C motif chemokine receptor 2 [Source:HGNC Symbol;Acc:HGNC:6027] | 9666 | -0.289 | -0.1948 | Yes |
| 174 | CCL2 | C-C motif chemokine ligand 2 [Source:HGNC Symbol;Acc:HGNC:10618] | 9819 | -0.303 | -0.2023 | Yes |
| 175 | IL18RAP | interleukin 18 receptor accessory protein [Source:HGNC Symbol;Acc:HGNC:5989] | 9832 | -0.304 | -0.1987 | Yes |
| 176 | IL3 | interleukin 3 [Source:HGNC Symbol;Acc:HGNC:6011] | 9850 | -0.306 | -0.1955 | Yes |
| 177 | CCL17 | C-C motif chemokine ligand 17 [Source:HGNC Symbol;Acc:HGNC:10615] | 9893 | -0.311 | -0.1941 | Yes |
| 178 | CXCL12 | C-X-C motif chemokine ligand 12 [Source:HGNC Symbol;Acc:HGNC:10672] | 9901 | -0.312 | -0.1900 | Yes |
| 179 | LTA | lymphotoxin alpha [Source:HGNC Symbol;Acc:HGNC:6709] | 9921 | -0.313 | -0.1869 | Yes |
| 180 | CXCR1 | C-X-C motif chemokine receptor 1 [Source:HGNC Symbol;Acc:HGNC:6026] | 9936 | -0.315 | -0.1832 | Yes |
| 181 | TNFRSF10D | TNF receptor superfamily member 10d [Source:HGNC Symbol;Acc:HGNC:11907] | 10055 | -0.326 | -0.1877 | Yes |
| 182 | TNFRSF9 | TNF receptor superfamily member 9 [Source:HGNC Symbol;Acc:HGNC:11924] | 10057 | -0.326 | -0.1829 | Yes |
| 183 | IL1R2 | interleukin 1 receptor type 2 [Source:HGNC Symbol;Acc:HGNC:5994] | 10262 | -0.347 | -0.1938 | Yes |
| 184 | TNFRSF12A | TNF receptor superfamily member 12A [Source:HGNC Symbol;Acc:HGNC:18152] | 10349 | -0.355 | -0.1953 | Yes |
| 185 | CCL8 | C-C motif chemokine ligand 8 [Source:HGNC Symbol;Acc:HGNC:10635] | 10529 | -0.376 | -0.2038 | Yes |
| 186 | OSMR | oncostatin M receptor [Source:HGNC Symbol;Acc:HGNC:8507] | 10560 | -0.379 | -0.2005 | Yes |
| 187 | TNFSF14 | TNF superfamily member 14 [Source:HGNC Symbol;Acc:HGNC:11930] | 10564 | -0.380 | -0.1951 | Yes |
| 188 | AMHR2 | anti-Mullerian hormone receptor type 2 [Source:HGNC Symbol;Acc:HGNC:465] | 10571 | -0.381 | -0.1899 | Yes |
| 189 | IL15RA | interleukin 15 receptor subunit alpha [Source:HGNC Symbol;Acc:HGNC:5978] | 10586 | -0.382 | -0.1853 | Yes |
| 190 | TNFRSF25 | TNF receptor superfamily member 25 [Source:HGNC Symbol;Acc:HGNC:11910] | 10646 | -0.387 | -0.1841 | Yes |
| 191 | TNFRSF10C | TNF receptor superfamily member 10c [Source:HGNC Symbol;Acc:HGNC:11906] | 10731 | -0.397 | -0.1848 | Yes |
| 192 | IFNB1 | interferon beta 1 [Source:HGNC Symbol;Acc:HGNC:5434] | 10801 | -0.406 | -0.1842 | Yes |
| 193 | IFNW1 | interferon omega 1 [Source:HGNC Symbol;Acc:HGNC:5448] | 10816 | -0.407 | -0.1793 | Yes |
| 194 | IL18R1 | interleukin 18 receptor 1 [Source:HGNC Symbol;Acc:HGNC:5988] | 10822 | -0.407 | -0.1736 | Yes |
| 195 | FLT1 | fms related receptor tyrosine kinase 1 [Source:HGNC Symbol;Acc:HGNC:3763] | 10826 | -0.408 | -0.1677 | Yes |
| 196 | IL19 | interleukin 19 [Source:HGNC Symbol;Acc:HGNC:5990] | 10846 | -0.410 | -0.1631 | Yes |
| 197 | CCL20 | C-C motif chemokine ligand 20 [Source:HGNC Symbol;Acc:HGNC:10619] | 10859 | -0.411 | -0.1579 | Yes |
| 198 | IL4R | interleukin 4 receptor [Source:HGNC Symbol;Acc:HGNC:6015] | 10902 | -0.417 | -0.1550 | Yes |
| 199 | BMP2 | bone morphogenetic protein 2 [Source:HGNC Symbol;Acc:HGNC:1069] | 10919 | -0.420 | -0.1500 | Yes |
| 200 | TNFRSF1B | TNF receptor superfamily member 1B [Source:HGNC Symbol;Acc:HGNC:11917] | 10969 | -0.427 | -0.1475 | Yes |
| 201 | ACVR2B | activin A receptor type 2B [Source:HGNC Symbol;Acc:HGNC:174] | 11011 | -0.431 | -0.1443 | Yes |
| 202 | TNFRSF10B | TNF receptor superfamily member 10b [Source:HGNC Symbol;Acc:HGNC:11905] | 11020 | -0.433 | -0.1384 | Yes |
| 203 | VEGFD | vascular endothelial growth factor D [Source:HGNC Symbol;Acc:HGNC:3708] | 11095 | -0.443 | -0.1377 | Yes |
| 204 | CCL16 | C-C motif chemokine ligand 16 [Source:HGNC Symbol;Acc:HGNC:10614] | 11140 | -0.449 | -0.1344 | Yes |
| 205 | BMPR2 | bone morphogenetic protein receptor type 2 [Source:HGNC Symbol;Acc:HGNC:1078] | 11174 | -0.454 | -0.1303 | Yes |
| 206 | CNTFR | ciliary neurotrophic factor receptor [Source:HGNC Symbol;Acc:HGNC:2170] | 11203 | -0.457 | -0.1256 | Yes |
| 207 | CCL4 | C-C motif chemokine ligand 4 [Source:HGNC Symbol;Acc:HGNC:10630] | 11211 | -0.458 | -0.1193 | Yes |
| 208 | INHBA | inhibin subunit beta A [Source:HGNC Symbol;Acc:HGNC:6066] | 11237 | -0.462 | -0.1144 | Yes |
| 209 | IL9 | interleukin 9 [Source:HGNC Symbol;Acc:HGNC:6029] | 11309 | -0.472 | -0.1130 | Yes |
| 210 | IL17B | interleukin 17B [Source:HGNC Symbol;Acc:HGNC:5982] | 11437 | -0.494 | -0.1156 | Yes |
| 211 | KDR | kinase insert domain receptor [Source:HGNC Symbol;Acc:HGNC:6307] | 11454 | -0.496 | -0.1095 | Yes |
| 212 | CCL3 | C-C motif chemokine ligand 3 [Source:HGNC Symbol;Acc:HGNC:10627] | 11465 | -0.497 | -0.1028 | Yes |
| 213 | KITLG | KIT ligand [Source:HGNC Symbol;Acc:HGNC:6343] | 11516 | -0.503 | -0.0993 | Yes |
| 214 | IL6R | interleukin 6 receptor [Source:HGNC Symbol;Acc:HGNC:6019] | 11615 | -0.519 | -0.0993 | Yes |
| 215 | EDA | ectodysplasin A [Source:HGNC Symbol;Acc:HGNC:3157] | 11897 | -0.571 | -0.1129 | Yes |
| 216 | LIF | LIF interleukin 6 family cytokine [Source:HGNC Symbol;Acc:HGNC:6596] | 12085 | -0.615 | -0.1185 | Yes |
| 217 | PDGFA | platelet derived growth factor subunit A [Source:HGNC Symbol;Acc:HGNC:8799] | 12104 | -0.621 | -0.1106 | Yes |
| 218 | IL1B | interleukin 1 beta [Source:HGNC Symbol;Acc:HGNC:5992] | 12105 | -0.622 | -0.1013 | Yes |
| 219 | EGFR | epidermal growth factor receptor [Source:HGNC Symbol;Acc:HGNC:3236] | 12109 | -0.622 | -0.0923 | Yes |
| 220 | IL13 | interleukin 13 [Source:HGNC Symbol;Acc:HGNC:5973] | 12173 | -0.636 | -0.0877 | Yes |
| 221 | ACVRL1 | activin A receptor like type 1 [Source:HGNC Symbol;Acc:HGNC:175] | 12242 | -0.656 | -0.0833 | Yes |
| 222 | CXCL8 | C-X-C motif chemokine ligand 8 [Source:HGNC Symbol;Acc:HGNC:6025] | 12297 | -0.676 | -0.0775 | Yes |
| 223 | CLCF1 | cardiotrophin like cytokine factor 1 [Source:HGNC Symbol;Acc:HGNC:17412] | 12360 | -0.695 | -0.0720 | Yes |
| 224 | IL3RA | interleukin 3 receptor subunit alpha [Source:HGNC Symbol;Acc:HGNC:6012] | 12380 | -0.703 | -0.0630 | Yes |
| 225 | PDGFB | platelet derived growth factor subunit B [Source:HGNC Symbol;Acc:HGNC:8800] | 12389 | -0.704 | -0.0531 | Yes |
| 226 | FLT4 | fms related receptor tyrosine kinase 4 [Source:HGNC Symbol;Acc:HGNC:3767] | 12396 | -0.705 | -0.0431 | Yes |
| 227 | IL1R1 | interleukin 1 receptor type 1 [Source:HGNC Symbol;Acc:HGNC:5993] | 12419 | -0.713 | -0.0341 | Yes |
| 228 | IL6 | interleukin 6 [Source:HGNC Symbol;Acc:HGNC:6018] | 12460 | -0.730 | -0.0264 | Yes |
| 229 | IL6ST | interleukin 6 cytokine family signal transducer [Source:HGNC Symbol;Acc:HGNC:6021] | 12505 | -0.749 | -0.0187 | Yes |
| 230 | CXCL3 | C-X-C motif chemokine ligand 3 [Source:HGNC Symbol;Acc:HGNC:4604] | 12516 | -0.756 | -0.0082 | Yes |
| 231 | VEGFA | vascular endothelial growth factor A [Source:HGNC Symbol;Acc:HGNC:12680] | 12629 | -0.813 | -0.0049 | Yes |
| 232 | INHBB | inhibin subunit beta B [Source:HGNC Symbol;Acc:HGNC:6067] | 12680 | -0.851 | 0.0039 | Yes |
| 233 | CXCL2 | C-X-C motif chemokine ligand 2 [Source:HGNC Symbol;Acc:HGNC:4603] | 12733 | -0.896 | 0.0132 | Yes |
Table: GSEA details [plain text format]

  

Fig 2: KEGG\_CYTOKINE\_CYTOKINE\_RECEPTOR\_INTERACTION      
 Blue-Pink O' Gram in the Space of the Analyzed GeneSet

  

Fig 3: KEGG\_CYTOKINE\_CYTOKINE\_RECEPTOR\_INTERACTION: Random ES distribution      
 Gene set null distribution of ES for **KEGG\_CYTOKINE\_CYTOKINE\_RECEPTOR\_INTERACTION**
